# Minds without borders: integrating social psychology into global health diplomacy

**DOI:** 10.3389/fpubh.2025.1667125

**Published:** 2025-10-02

**Authors:** Nadine Knab, Malek Bajbouj

**Affiliations:** Klinik für Psychiatrie und Psychotherapie, Einstein Research Unit Coping with Affective Polarization, Charite - Universitatsmedizin Berlin, Berlin, Germany

**Keywords:** health diplomacy, social psychology, global health, contact, identity, norms, social identity theory, contact hypothesis

In addressing the escalating challenges of global health crises, we propose a conceptual model integrating key insights from social psychology into the practice of health diplomacy. This lens allows to understand and navigate the human dimensions of global health efforts, emphasizing the importance of integrating social-psychological knowledge into global health efforts. By focusing on contact, identity, and norms, it allows examination how these elements influence behaviors and partnerships in global health. Global mental health is increasingly recognized as a critical component of public health, influencing not only individual wellbeing but also societal stability and international relations (1, 2). Mental health challenges—exacerbated by conflicts, displacement, climate change, and global health crises—transcend national borders that require coordinated efforts across countries. In addition, mental health can pose a serious pre-condition for transgenerational violence. Augsburger and Jacob (3) discuss how trauma, particularly post-traumatic stress disorder (PTSD), contributes to cycles of violence, especially in conflict and crisis regions. PTSD can perpetuate aggression, which complicates reconciliation processes, as former victims often become perpetrators. Effective reconciliation and stabilization must include mental health recovery programs to break violence cycles, reduce stigmatization, and facilitate reintegration and reconciliation, emphasizing the need for community-based mental health structures. This urgency places mental health at the forefront of health diplomacy—that is, the international and cross-sector collaboration between governments, organizations and communities aimed at jointly addressing global health challenges. Health diplomacy holds particular significance in *track 2* and *track 3 diplomacy*—terms commonly used to describe informal channels of international engagement that complement official state-led negotiations (*track 1 diplomacy*). Whereas, track 1 diplomacy involves formal interactions between government representatives, track 2 diplomacy refers to semi-official dialogues and collaborative efforts among non-state but influential actors, such as academics, policy experts, former officials and thought leaders. These actors engage in dialogue processes aimed at reducing tensions and shaping policy options outside the constraints of official political positions. *Track 3 diplomacy*, in contrast, focuses on even more grassroots-level efforts. It involves the engagement of civil society actors, including non-governmental organizations (NGOs), faith-based groups, community leaders, and activists. These initiatives seek to build trust, promote intercultural understanding and foster peace through community-driven approaches. In the context of global health, track 2 and 3 diplomacy play essential roles by enabling cooperation on shared health challenges, especially in politically sensitive contexts where official diplomatic relations may be strained or absent.

Unlike traditional diplomatic efforts that often focus directly on resolving political or economic disparities between groups, health diplomacy operates as an indirect intervention. It does not directly address group disparities but can still exert considerable influence by leveraging the interconnected nature of global health challenges. At its core, social psychology is the study of how individuals' thoughts, feelings, and behaviors are influenced by their social environments (4). It explores phenomena such as group dynamics, prejudice, identity formation and social norms, all of which are relevant to global mental health and health diplomacy efforts. Social psychology offers tools to understand how stigma is perpetuated, how intergroup conflicts arise, and how collective actions are mobilized. These insights are critical for health diplomacy, where success often depends on navigating complex interpersonal and intergroup dynamics. By integrating socio-psychological principles, health diplomacy can move beyond top-down solutions that usually directly address conflict by leveraging on important socio-psychological principles. We will now expand on a selection of these principles below.

## Social identity: channeling group dynamics for global health

Social identity theory (5) offers valuable insights into how group membership shapes behavior, attitudes and perceptions. Individuals derive a sense of self from their group identities, which can either foster inclusion and solidarity or exacerbate divisions (6). This dynamic is especially relevant in the context of global health, where stigma, exclusion and inequality often undermine access to care (7). Recognizing and affirming cultural and social identities can foster trust and engagement, while promoting shared identities—such as a global health identity—can unite disparate groups under a common purpose (8, 9). For example, framing climate change's health impacts as a collective challenge could inspire cross-border solidarity and cooperative action (10, 11). Promoting a shared identity could also help reduce stigma around mental health by encouraging the recognition of psychological wellbeing as a universal concern rather than an individual weakness or taboo. Selecting a shared identity frame is especially difficult in intractable conflicts marked by collective—rather than merely individual—trauma which impacts capacities for peace processes (12). Competitive victimhood—an intergroup dynamic in which each side seeks recognition as the “true” or greater victim to gain moral standing and justification—hardens group boundaries and undermines superordinate identity-building based on social groups (13); by contrast, health-anchored identities are often less politicized and could offer a more unifying basis for cooperation.

## The contact hypothesis: building trust through interaction

The contact hypothesis (14) emphasizes the potential of intergroup contact to reduce prejudice and foster trust. When individuals from different groups interact under conditions of equality, shared goals, cooperation and institutional support, they are more likely to overcome biases and build mutual understanding [the contact situation can still have a positive impact even if not all conditions are met see (15)]. Within health diplomacy, this principle can guide initiatives that bring together diverse stakeholders—nations, communities and organizations—to collaborate on shared health challenges. For instance, during vaccine distribution campaigns, fostering contact between international health teams and local leaders has been shown to build trust, reduce resistance, and enhance the perceived legitimacy of interventions (16). Thus, a deliberate use of such contact opportunities could strengthen cooperation in health efforts. Project Rozana—an international organization connecting Israeli and Palestinian health care professionals—shows how their programs built on contact advanced cross-border collaboration (17). Health diplomacy efforts can also function as an indirect contact intervention by fostering extended contact between groups through collaborative health initiatives (18, 19). Extended contact theory posits that observing positive interactions between members of different groups—such as through partnerships in health programs—can reduce prejudice and promote intergroup understanding without direct personal interaction. While extended contact tends to be less impactful than direct interactions (39), results from a meta-analysis suggest that it can produce small to medium positive effects on intergroup attitudes (20). Health diplomacy initiatives that bring together representatives from diverse cultural, social, or political backgrounds to address shared health challenges can create opportunities for symbolic and vicarious contact. These efforts could promote narratives of cooperation, mutual respect, and trust, which can shift perceptions, challenge stereotypes, and influence broader social norms. By strategically highlighting these success stories and amplifying their visibility, health diplomacy can create a ripple effect, encouraging further collaboration and reducing tensions in other domains (21). Leveraging this form of extended contact within global health efforts can enhance their impact by not only addressing health disparities but also fostering greater social cohesion and solidarity across divided groups. Contact interventions have also received criticism by potentially producing an “irony of harmony,” in which power asymmetries are perceived as less salient (22, 23). Embedding structured perspective-giving—especially prioritizing voice for disadvantaged groups—into health-diplomacy contact programs can correct status asymmetries, build felt respect and mutual trust, and translate into more durable cooperation [(24) see also (25)]. Thus, the methods and tools need to be embedded in the broader socio-psychological context, in which for example norms play an important role.

## Emphasizing equal partnerships and cooperation or maintaining inequalities?

Research in social psychology highlights that helping behaviors are often shaped by implicit norms about power and dependency (26). Dependency-oriented helping refers to a form of assistance in which support is provided in a way that reinforces the recipient's reliance on the helper, limiting opportunities for autonomy and capacity-building. In the context of global mental health, this can occur when interventions are designed and delivered by external actors without engaging local communities, professionals, or cultural knowledge systems. For example, when international organizations implement mental health programs in low- and middle-income countries using imported models and foreign personnel—without training local providers or adapting to local contexts—communities may remain dependent on external aid. While such efforts may offer short-term relief, they risk undermining local ownership, sustainability and trust and may ultimately reinforce systemic inequalities in global health governance. Thus, dependency-oriented helping, where the helper maintains control and limits the recipient's autonomy, could perpetuate inequalities and undermine trust. For example, a real-world case study of health organizations in Malawi showed how limited discretion over aid and donor-driven control created a cycle of mistrust and diminished local capacity, undermining cooperation and health outcomes [but see bidirectional impact of trust on power outline in (27)]. In global health contexts, such as delivering aid to marginalized communities, this type of helping can reinforce stigma and disempower the very groups it aims to support (28, 29). To exemplify, during the COVID-19 pandemic, high-income countries were investing in securing high amounts of the vaccines and later shared them for reasons of solidarity, but it could be seen as means to assert a geo-political advantage (30). In addition, people receiving dependency-oriented help infer status maintaining motivations from the help provider—which could then further stain cooperative relations as the help recipients tend to show lower levels of cooperation intention in return (31). Especially in conflict-torn contexts or contexts with a long history of intergroup conflicts, there might be a high level of threat perception. When dominant groups perceive others as threats—whether due to cultural differences, resource competition, or political instability—these perceptions can manifest in paternalistic helping behaviors (32, 33). For example, aid programs may focus on alleviating immediate symptoms without addressing systemic inequities, reinforcing a sense of dependency. Social norms play a crucial role in shaping how such programs operate and influence behavior, making them highly relevant for promoting social change (34, 35). But recent research indicates an interplay between subjective social norms and threat perceptions: when threat perceptions are coupled with strong perceived social norms to help (for example by global health initiatives with institutional support), this could lead to an especially high level of dependency-oriented helping (36). This dynamic is particularly complex in the global health context, where norms embedded in health interventions often reflect Western values and assumptions. The export of these norms can unintentionally undermine local knowledge systems and community autonomy. This is especially evident in the realm of mental health, where Western norms tend to emphasize individual pathology, clinical diagnoses and formal treatment, while in many other cultural contexts, mental distress may be interpreted through social, spiritual, or communal lenses. Imposing Western conceptualizations of mental illness may not only lead to culturally incongruent interventions but also risk stigmatizing local coping strategies and support systems. As a result, well-intentioned efforts to promote health and wellbeing may contribute to disempowerment, reinforce unequal power dynamics and inhibit sustainable, locally driven solutions. A critical reflection on which norms are being promoted and whose voices are shaping them, is thus essential to ensure that global health efforts support rather than compromise empowerment and equity. Past research has also shown that reconciliation is enhanced when interventions “speak” to peoples' specific needs. In detail, perpetrators and victims have different underlying needs and acknowledging them before bringing the past adversary groups together could have a beneficial impact (37). In summary, the concrete way of interacting and cooperation needs to be reflected upon, so that initiatives can lead to the empowerment of communities and thus strengthen the trust between the involved parties. For instance, research indicates that asking recipients how they would like to be helped reduces the likelihood of offering dependency-oriented assistance. These findings highlights the importance of involving both help providers and recipients equally in the process (38).

## Integrating contact and identity for health diplomacy

**Figure 1:**
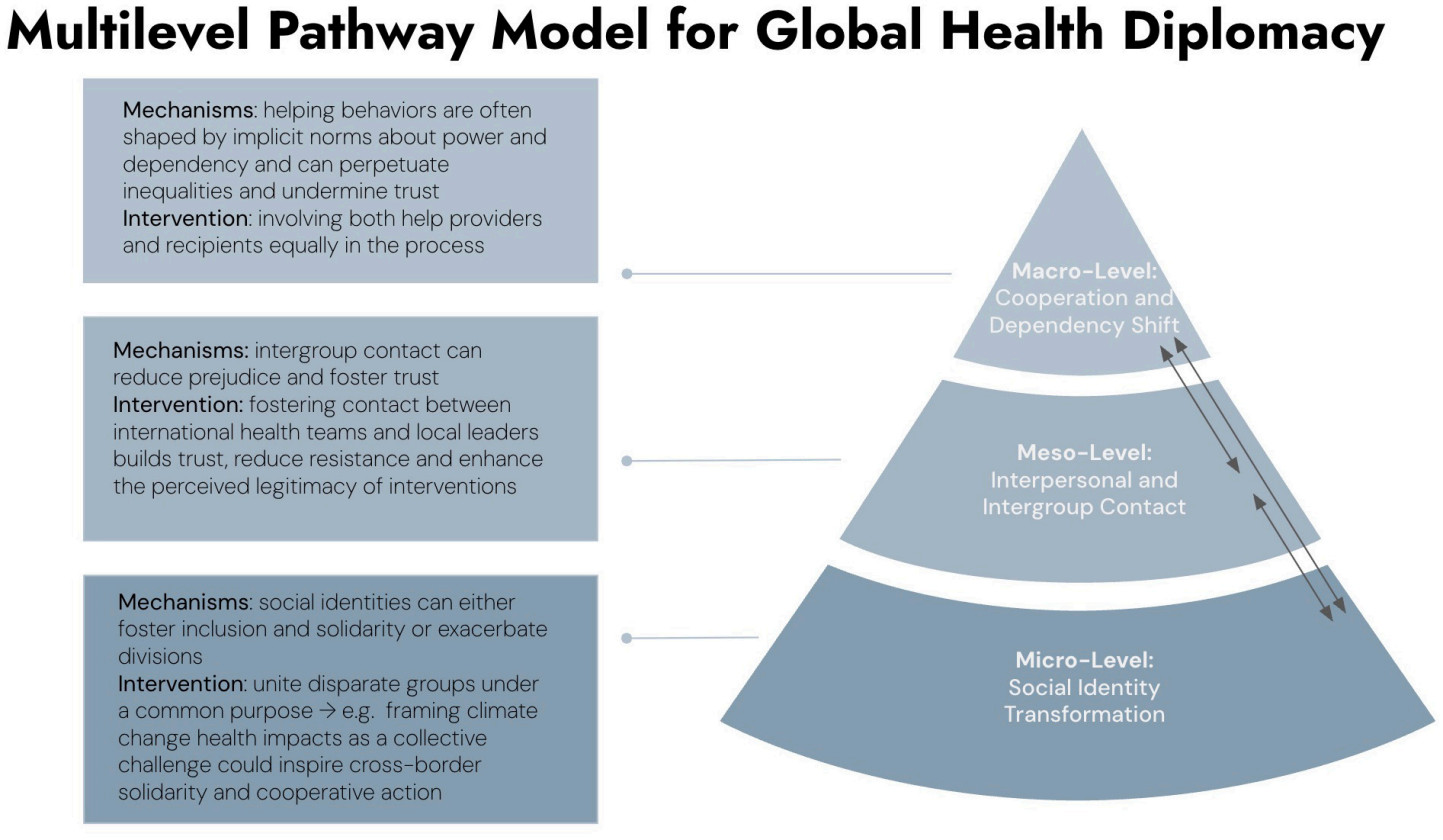
Multilevel pathway model for global health diplomacy.

By integrating the contact hypothesis and social identity theory, this approach addresses both interpersonal and group-level dynamics. Key applications include (see Figure 1):

1. Promoting Shared Goals: Diplomatic initiatives can frame global health challenges as shared threats, fostering a collective “global health” identity—but employ culturally sensitive communication that respects existing identities while highlighting collective goals to bridge divides.
2. Facilitate contact: Structured opportunities for intergroup contact—such as cross-border health collaborations—can reduce biases and perceived status differences, particularly when all parties are seen as equal contributors.
3. Redefining Norms: Before initiating interventions, conduct detailed assessments of local norms around mental health, wellbeing, and social support practices.
4. Establish inclusive Partnerships: Equal partnerships help establish new norms of cooperation and reciprocity, moving away from the implicit hierarchies that often characterize global health efforts. This could be by dedicating a portion of project budgets specifically to training healthcare workers in mental health care, conflict resolution, and culturally adapted treatment practices—designed as a bilateral exchange in which both partners share and develop skills and competencies.

## Conclusion

We propose that a socio-psychological lens is beneficial for health diplomacy efforts and can address both the technical and social dimensions of global health challenges to contribute to transcending divisions and promote trust and cooperation.
